# Development of an Age-Appropriate Household Dysfunction Measure and its Concurrent Validity With Multiple Outcomes Among Middle School Adolescents in Southeast Texas

**DOI:** 10.1177/08862605251341285

**Published:** 2025-05-29

**Authors:** Sumaita Choudhury, Timothy J. Walker, Emily T. Hébert, Christine M. Markham, Ross Shegog, Robert C. Addy, Melissa F. Peskin

**Affiliations:** 1 The University of Texas Health Science Center at Houston (UTHealth Houston) School of Public Health, USA; 2 The University of Texas Health Science Center at Houston (UTHealth Houston) School of Public Health, Austin, USA

**Keywords:** household dysfunction, adverse childhood experiences, concurrent validity, dating violence, substance misuse, mental health, adolescents

## Abstract

Existing adverse childhood experience (ACE) measures include a limited number of household dysfunction (HD) items and the use of adult-oriented language that is not always appropriate for middle schoolers. We developed an age-appropriate 10-item HD measure informed by previously validated ACE measures and tested its concurrent validity with dating violence (DV) perpetration, lifetime substance use, and mental health outcomes among middle schoolers in Southeast Texas. This cross-sectional study used the baseline data from a DV prevention intervention program for sixth graders (*N* = 126), Me & You Tech. Baseline data were collected from March 2023 to April 2023. To test the concurrent validity of the newly developed HD measure, we conducted a series of multivariable regression models regarding the association between HD and the six key outcomes while controlling for covariates. The most common type of HD exposure was parental separation/divorce (33.3%). We found significant associations between HD and physical (AOR = 1.45; 95% CI [1.00, 2.10]) and psychological DV perpetration (AOR = 1.75; 95% CI [1.20, 2.56]). For each additional reported HD exposure, there were 61% higher odds for adolescents to engage in lifetime alcohol use (AOR = 1.61; 95% CI [1.11, 2.34]). Finally, we found significant associations between HD and depression (β = 1.34; 95% CI [0.57, 2.12]) and HD and anxiety (β = 1.28; 95% CI [0.52, 2.03]). Our findings suggest the HD measure is pragmatic and has strong evidence of concurrent validity. This measure may be a helpful tool in assessing HD among middle school-aged adolescents, both in school and clinic settings.

## Introduction

Adverse childhood experiences (ACEs) are traumatic events that occur before the age of 18, such as physical and sexual abuse, physical and emotional neglect, and household dysfunction (HD; CDC, 2022a). HD, one of the domains of ACEs, has been defined as children living in unstable households witnessing intimate partner violence, parental separation, family loss, household member(s) with mental health and/or substance use disorders, household member(s) with a disability, household food insecurity, experiencing homelessness, and family history of incarceration (Afifi, Salmon, et al., 2020; Afifi, Taillieu, et al., 2020; Bussemakers et al., 2022; Felitti et al., 1998). HD has been shown to have detrimental effects on children’s behavioral and mental health-related outcomes. For example, HD has been linked with dating violence (DV) perpetration, substance use, anxiety, and depression among adolescents and young adults (Calvete et al., 2016; Duke et al., 2010; Ehrensaft et al., 2003; Herrenkohl et al., 2004; Jouriles et al., 2012; Linder & Collins, 2005; McCoy et al., 2020; Morris et al., 2015; Negriff, 2020; Reyes et al., 2015; Stewart et al., 2022). Despite HD’s adverse effects, most research has focused on adults (Dube et al., 2003; Navarro et al., 2022; Negriff, 2020). More research is necessary to better understand how HD impacts younger age groups, particularly middle school-aged (11–14 years) adolescents.

To conduct this research, there is a need for HD-specific measures that are age-appropriate for middle school adolescents. However, existing ACE measures were initially designed for adults and older adolescents who are primarily assessed in clinical settings (Afifi, Salmon, et al., 2020; Afifi, Taillieu, et al., 2020). These ACE measures use adult-oriented language, which is not always appropriate for middle school adolescents. Furthermore, most adult ACE measures include questions with multiple concepts regarding trauma within a single item. For example, one ACE item asks whether individuals experienced food insecurity or whether their parents were alcoholics (Felitti et al., 1998). Including multiple concepts within a single item can make it challenging to assess which exposure contributed to adverse outcomes. In addition, adult ACE measures prompt individuals to recall and report their ACEs that occurred during childhood, which requires items to be rephrased when used among younger populations. Although some specific ACE measures were developed for older adolescents (i.e., the Pediatric ACEs and Related Life Events Screener [PEARLS]), these items still lack age-appropriateness for younger ages and can cause administration issues due to the mature language and legal reporting requirements in certain states (Bernstein et al., 1994; PEARLS, 2019).

Moreover, most ACE measures were designed as composite measures rather than domain-specific measures. This means the measures encompass multiple domains with a limited number of items for each respective domain. Composite measures are valuable for assessing the broader effect of ACEs on different outcomes (McLennan et al., 2020; Mersky et al., 2017). However, they lack the specificity to adequately examine the unique impact of each domain, such as HD.

Another measurement challenge is that the existing ACE measures use different questions to assess respective domains. Although there are several versions of the ACE measures (such as the Behavioral Risk Factor Surveillance System [BRFSS], National Survey of Children’s Health [NSCH], and PEARLS), they include a limited number of domain-specific items that are inconsistent between measures (BRFSS, 2021; NSCH, 2021; PEARLS, 2019; Spinhoven et al., 2014; Walsh et al., 2008). For example, PEARLS includes HD items related to homelessness and food insecurity, which are not included in BRFSS and NSCH (BRFSS, 2021; NSCH, 2021; PEARLS, 2019). Moreover, psychometric testing of existing ACE measures has revealed inconsistent evidence regarding the type and number of items included under the HD subscales/domains (Afifi, Salmon, et al., 2020; C. Choi et al., 2020; Ford et al., 2014; Mersky et al., 2017; Schnarrs et al., 2022). The subscale-related inconsistencies may be due to domain-specific measure discrepancies, leading to inadequate assessment of the domains. Due to these measurement-related issues, there is a need for improved measures focusing on HD for middle schoolers.

Enhancing the measurement of HD for middle schoolers is essential for improving our understanding of how HD (compared to other ACE domains) may differentially impact behavioral and health outcomes. In addition, a new enhanced age-appropriate HD measure that captures different aspects of HD will provide better insights regarding the adverse consequences of HD, help intervene earlier to provide support, and inform efforts for future interventions for middle schoolers. Therefore, the purpose of the study was to develop an age-appropriate HD measure, informed by previously validated composite ACE measures. In addition, we tested the concurrent validity of the HD measure by examining its association with DV perpetration, lifetime substance use, and mental health among middle schoolers in Southeast Texas.

## Methods

### Study Design and Data Collection

We used the baseline data from the evaluation of Me & You Tech (MyTech), a multi-level computer-based dating violence prevention program for sixth-grade adolescents in Southeast Texas (Peskin et al., 2019). We employed a cross-sectional design to evaluate the concurrent validity of the HD measure concerning six outcomes, including physical and psychological DV perpetration, lifetime alcohol and drug use, and anxiety and depression among middle school adolescents.

Testing the concurrent validity provides evidence about whether the newly developed HD measure captures the construct as intended by exhibiting the expected associations with relevant outcome variables (Bandalos, 2018). We selected these outcome variables based on previous empirical evidence (Chatterjee et al., 2018; Foshee et al., 2015; Gottfredson et al., 2022; McCoy et al., 2020; Ontiveros et al., 2020; Reyes et al., 2015; Temple et al., 2013; Wang et al., 2021). Baseline data were collected from March 2023 to April 2023. Parental consent and student assent forms were obtained before the dissemination of the surveys. The study was approved by the Institutional Review Board at the UTHealth Science Center in Houston (HSC-SPH-19-0253, May 02, 2019).

### Eligibility Criteria

Adolescents in sixth grade, between the ages of 11 to 14 years, and currently enrolled at a participating middle school were eligible for the study.

### Measures

#### Development of HD Measure Items

We developed the 10-item *HD measure* using a four-step approach informed by the DeVellis scale development guidelines (Supplemental Material 1; DeVellis, 2011). First, we defined the HD domain based on previous relevant studies that assessed HD and generated a definition that captured the construct adequately. Second, we created items for the HD measure by reviewing existing ACE measures and psychometric literature that identified HD as a subscale. After a thorough review, we identified five HD items from the BRFSS ACE measure, two items from the NSCH ACE measure, and three from the PEARLS measure to include in the pool of potential items. As our study population is middle schoolers, we adapted the items by rephrasing the existing language of all HD items to be age-appropriate for sixth graders. In addition, we modified the tense of all items, from past to present, to account for current HD exposures, sensitivity, and reporting law issues relevant to this population.

For the third step, we designated dichotomized (*yes*/*no*) response options for our HD measure, as the items are phrased straightforwardly, requiring simple dichotomized response options to be the optimal choice. Fourth, we reviewed the new set of items, and a content expert (JT) also reviewed the measure for face validity, which led to refinements of item phrasing to ensure readability and clarity. The research team reviewed the item changes and reached a consensus for the final set of ten items to include in the HD measure. Finally, consistent with previous studies, a cumulative HD score was established by adding the total number of exposures with scores ranging from 0 to 10, with 0 being no exposure to HD and 10 being extreme exposure to HD (Dong et al., 2004; Luft et al., 2022; Musa et al., 2018; Schuler et al., 2021).

### Concurrent Validity Variables

#### DV Perpetration

DV perpetration refers to committing various forms of intimate partner violence, including physical, psychological, sexual, threatening, digital, or stalking during a romantic relationship (CDC, 2022b). For this study, we used the *physical and psychological subscales* from the Conflict in Adolescent Dating and Relationship Inventory (CADRI) to assess DV perpetration (Supplemental Material 2; Wolfe et al., 2001). Physical DV perpetration was assessed by four items (e.g., “I threw something at him/her”), and psychological DV perpetration was measured by 13 items (e.g., “I did something to make him/her jealous”) with dichotomized response options “*yes*” or “*no*.”

Adolescents who have participated in one or more items related to physical DV perpetration were coded as “*yes*” for perpetrators. Adolescents who did not participate in DV perpetration or never dated (i.e., having a boyfriend or girlfriend) or did not have dating partners in the past 12 months were coded as “*no*” for non-DV perpetrators (Peskin et al., 2019). The psychological DV subscale was scored similarly. However, three or more indications of psychological DV perpetration were coded as “*yes*” (H. J. Choi et al., 2017; Peskin et al., 2019).

#### Life-Time Substance Use

Lifetime *alcohol use* was assessed using a single item, “*Have you ever had a drink of alcohol (other than a few sips)? (When thinking about ‘drinking alcohol’, please think in terms of any alcoholic beverage such as beer, wine, whiskey, liquor, rum, scotch, vodka, gin, or various alcoholic mixed drinks. Please also keep in mind that one drink of alcohol refers to one beer, one shot of liquor, or one glass of wine)*” with a binary response option “*yes*” or “*no*” (YRBS, 2023).

Lifetime *drug use* was measured by a single item asking adolescents to indicate if they have ever used any of the following drugs in their lifetime. The list of responses includes cigarettes, electronic cigarettes, chewing tobacco, snuff or dip, other smoking tobaccos, marijuana, synthetic marijuana, cocaine, amphetamines, inhalants, hallucinogens, over-the-counter cold or cough medicine with the intent of getting high, ecstasy, prescription medications that weren’t prescribed by a health professional, or other. The responses were combined into a binary variable where the selection of any type of drugs from the list was coded as “*yes*” and the response “*no*” was coded for adolescents who did not select any drugs (YRBS, 2023).

#### Mental Health Outcomes

*Depression* was assessed by a 6-item measure utilizing a 5-Point Likert scale (1–*never*, 2–*seldom*, 3–*sometimes*, 4–*often*, and 5–*always*; Supplemental Material 3). This measure consists of items related to sadness, mood, hopelessness, eating behaviors, sleep activity, and concentration. We used summative scoring for the depression measure, with a total score ranging from 6 to 30 (Dahlberg et al., 2005; Orpinas, 1993).

*Generalized anxiety* (GAD-7) was evaluated by a seven-item measure using a 4-Point Likert scale ranging from 0 (*not at all*), 1 (*several days*), 2 (*more than half the days*), and 3 (*nearly every day*; Supplemental Material 4). The measure includes items related to various facets of anxiety, including feeling on edge, constant worrying, worrying about different things, being unable to relax, restlessness, being easily irritated, and feeling scared about the future. We used summative scoring for the anxiety measure, with a total score ranging from 0 to 21 (Spitzer et al., 2006).

#### Covariates

Based on empirical evidence, (Afifi, Salmon, et al., 2020; Crouch et al., 2019; Peskin et al., 2019) the present study adjusted for the following covariates: age (11–14 years old), gender identity (girl, boy, or prefer to self-describe), race and ethnicity combined into a composite variable and recoded into three categories: African American, Hispanic, and Another Race (which includes American Indian or Alaskan Native, Native Hawaiian or Other Pacific Islander, Asian, Multiracial or mixed race, White or Other), and sexual orientation recoded into a dichotomized variable (gay/lesbian/bisexual/other or heterosexual).

#### Statistical Analysis

All statistical analyses were conducted using STATA/BE software version 17.0 (StataCorp, LLC, 2021). First, we evaluated the descriptive statistics of our sample characteristics. Specifically, for the categorical variables (i.e., gender, race, ethnicity, and sexual orientation), we reported the frequencies and percentages. Conversely, we reported the mean and standard deviation for the continuous variable (i.e., age). Second, we examined the descriptive statistics of the HD measure items and reported the frequencies and percentages to identify the most common types of HD exposures in our study sample.

Third, we conducted bivariate models to examine the independent associations between HD and the six outcomes. Therefore, we first conducted simple logistic regression models to evaluate the association between HD and the four binary outcomes (i.e., physical and psychological DV perpetration and lifetime alcohol use and drug use). Second, we employed simple linear regression models to examine the association between HD and the two continuous outcomes (i.e., depression and anxiety).

To further assess the concurrent validity, we conducted a series of multivariable regression models while controlling for covariates. First, we conducted logistic regression models to examine the association between HD and the four binary outcomes. Next, we employed linear regression models for the two continuous mental health outcomes. We also examined the Intraclass Correlation Coefficients (ICCs) for potential clustering effects but found them to be below 0.01.

## Results

### Study Sample Characteristics

Overall, 126 adolescents with a mean age of 11.8 (±0.7) years participated in the Me & You Tech baseline survey (Table 1). Fifty percent of the sample identified as boys. Regarding race and ethnicity, 53.2% of the sample reported being Hispanic, 32.5% reported being African American, and 12.7% reported to be Another Race, which includes (American Indian or Alaskan Native, Native Hawaiian or Other Pacific Islander, Asian, Multiracial, or mixed race, White or Other). In the context of sexual orientation, 74.6% of adolescents self-identified as heterosexual and 23% identified as gay, lesbian, bisexual, or other (i.e., adolescents who responded as “*not sure*” or “*something else*”).

**Table 1.** Descriptive Statistics of Study Sample.

Variables	*n* ^d^	%
Gender^a^		
Girl	63	50.0
Boy	63	50.0
Race/Ethnicity^b^		
African American	41	32.5
Hispanic	67	53.2
Another race	16	12.7
Sexual orientation^c^		
Gay/lesbian/bisexual/other	29	23.0
Heterosexual	94	74.6
	*n*	*M* (*SD*)
Age	125	11.8 (0.7)

*Note*. M = mean; SD = standard deviation; n = number of individuals.

a Gender also included a third category labeled as “prefer to self-describe,” which received zero responses.

b Another Race under race/ethnicity category includes (American Indian or Alaskan Native, Native Hawaiian or Other Pacific Islander, Asian, Multiracial, or mixed race, White or Other).

c Other under sexual orientation category includes (not sure yet and something else).

d Sample size varies due to missing data for race/ethnicity, sexual orientation, and age, which were less than 2.5%.

### HD Measure Descriptive Statistics

The cumulative HD score in our study population ranged from 0 to 7 (Table 2). Overall, 31% of our study population reported no HD exposure(s), and 28.6% of our sample reported being exposed to at least one type of HD. The most common type of HD exposure in our sample was parental separation or divorce (33.3%), followed by 17.5% who reported a deceased parent/guardian.

**Table 2.** Descriptive Statistics of Household Dysfunction Measure (*N* = 126).

HD Items	Yes	No	Missing
	*n* (%)	*n* (%)	*n* (%)
1. Do you live with anyone who is sad the majority of the time?	20 (15.9)	104 (82.5)	2 (1.6)
2. Do you live with anyone who drinks too much?	11 (8.7)	112 (88.9)	3 (2.4)
3. Do you live with anyone who abuses drugs or their medications?	6 (4.8)	116 (92.1)	4 (3.2)
4. Do you live with anyone who went to jail or is currently in jail?	20 (15.9)	103 (81.8)	3 (2.4)
5. Are your parents separated or divorced?	42 (33.3)	80 (63.5)	4 (3.2)
6. Do you live with anyone who says mean things or hurts other members of your house?	19 (15.1)	104 (82.5)	3 (2.4)
7. Have you lived with a parent or guardian who died?	22 (17.5)	100 (79.4)	4 (3.2)
8. Is your family having problems with stable housing? (e.g., not having a permanent place to live, having to move often, or having to live with multiple family members).	8 (6.4)	115 (91.3)	3 (2.4)
9. Do you often worry that you do not have enough food to eat at home?	16 (12.7)	107 (84.9)	3 (2.4)
10. Do you live with anyone who has a serious physical illness or disability?	16 (12.7)	106 (84.1)	4 (3.2)

*Note*. Items 1 to 5 were sourced from BRFSS, items 6 to 7 were obtained from NSCH, and items 8 to 10 were acquired from PEARLS.

### DV Perpetration

Results from the bivariate models indicated significant associations between HD and physical DV perpetration (OR = 1.38; 95% CI [1.01, 1.90]), and HD and psychological DV perpetration (OR = 1.64; 95% CI [1.19, 2.24]; Table 3). The multivariable results were consistent with bivariate results indicating that for each additional reported HD exposure, there were 45% higher odds for adolescents to engage in physical DV perpetration (AOR = 1.45; 95% CI [1.00, 2.10]) and 75% higher odds to perform psychological DV perpetration (AOR = 1.75; 95% CI [1.20, 2.56]) when holding other variables constant.

**Table 3.** Simple and Multivariable Logistic Regression Regarding the Association Between Household Dysfunction and Dating Violence Perpetration and Covariates.

Variables	Physical DV				Psychological DV			
	Bivariate		Multivariable		Bivariate		Multivariable	
	OR	95% CI	AOR	95% CI	OR	95% CI	AOR	95% CI
Household dysfunction	1.38*	1.01, 1.90	1.45*	1.00, 2.10	1.64**	1.19, 2.24	1.75**	1.20, 2.56
Age	1.12	0.50, 2.49	1.27	0.50, 3.23	1.56	0.75, 3.26	2.23	0.85, 5.80
Gender								
Girl	Ref.	Ref.	Ref.	Ref.	Ref.	Ref.	Ref.	Ref.
Boy	0.75	0.24, 2.31	0.54	0.13, 2.19	0.58	0.20, 1.70	0.38	0.09, 1.66
Race/Ethnicity								
African American	Ref.	Ref.	Ref.	Ref.	Ref.	Ref.	Ref.	Ref.
Hispanic	0.39	0.12, 1.34	0.34	0.09, 1.32	0.26*	0.07, 0.94	0.16*	0.03, 0.74
Another race	0.67	0.12, 3.65	0.21	0.02, 2.22	1.33	0.34, 5.25	0.44	0.07, 2.64
Sexual orientation								
Gay/lesbian/bisexual/other	Ref.	Ref.	Ref.	Ref.	Ref.	Ref.	Ref.	Ref.
Heterosexual	0.69	0.20, 2.45	0.93	0.22, 3.96	0.43	0.14, 1.32	0.69	0.17, 2.76

OR = unadjusted odds ratio; AOR = adjusted odds ratio; 95% CI = 95% confidence interval; Ref. = reference group. Sample sizes vary due to missing data.

Statistical significance at *p < .05. **p < .01.

### Lifetime Substance Use

The bivariate results demonstrated a significant association between HD and lifetime alcohol use (OR = 1.56; 95% CI [1.13, 2.17]; Table 4). The multivariable results exhibited that for each additional reported HD exposure, there were 61% higher odds for adolescents to engage in lifetime alcohol use (AOR = 1.61; 95% CI [1.11, 2.34]) when holding other variables constant. The findings from the bivariate and adjusted model revealed a nonsignificant association between HD and lifetime drug use.

**Table 4.** Simple and Multivariable Logistic Regression Regarding the Association Between Household Dysfunction and Lifetime Substance Use and Covariates.

Variables	Alcohol Use				Drug Use			
	Bivariate		Multivariable		Bivariate		Multivariable	
	OR	95% CI	AOR	95% CI	OR	95% CI	AOR	95% CI
Household dysfunction	1.56**	1.13, 2.17	1.61*	1.11, 2.34	1.32	0.95, 1.85	1.34	0.92, 1.94
Age	1.54	0.69, 3.44	1.10	0.36, 3.36	1.02	0.48, 2.18	1.34	0.58, 3.09
Gender								
Girl	Ref.	Ref.	Ref.	Ref.	Ref.	Ref.	Ref.	Ref.
Boy	1.69	0.52, 5.48	6.59*	1.06, 40.99	0.72	0.25, 2.08	0.57	0.16, 2.00
Race/Ethnicity								
African American	Ref.	Ref.	Ref.	Ref.	Ref.	Ref.	Ref.	Ref.
Hispanic	0.97	0.22, 4.29	1.25	0.24, 6.55	0.43	0.14, 1.35	0.41	0.12, 1.39
Another Race	4.00	0.78, 20.48	2.11	0.30, 15.01	0.67	0.12, 360	0.27	0.03, 2.67
Sexual orientation								
Gay/lesbian/bisexual/other	Ref.	Ref.	Ref.	Ref.	Ref.	Ref.	Ref.	Ref.
Heterosexual	0.32	0.10, 1.03	0.23	0.04, 1.23	0.91	0.27, 3.09	1.11	0.27, 4.56

*Note*. OR = unadjusted odds ratio; AOR = adjusted odds ratio; 95% CI = 95% confidence interval; Ref. = reference group. Sample sizes vary due to missing data.

Statistical significance at *p < .05. **p < .01.

### Mental Health Outcomes

The bivariate results indicated a significant association between HD and depression (*b* = 1.67; 95% CI [0.93, 2.40]) and HD and anxiety (*b* = 1.86; 95% CI [1.10, 2.61]) among adolescents (Table 5). Results from the adjusted model also revealed significant associations between HD and depression (β = 1.34; 95% CI [0.57, 2.12]) and HD and anxiety (β = 1.28; 95% CI [0.52, 2.03]).

**Table 5.** Simple and Multivariable Linear Regression Regarding the Association Between Household Dysfunction and Mental Health and Covariates.

Variables	Depression				Anxiety			
	Bivariate		Multivariable		Bivariate		Multivariable	
	Coef.	95% CI	β	95% CI	Coef.	95% CI	β	95% CI
Household dysfunction	1.67***	0.93, 2.40	1.34***	0.57, 2.12	1.86***	1.10, 2.61	1.28***	0.52, 2.03
Age	0.08	–1.72, 1.88	–0.22	–1.96, 1.51	0.24	–1.59, 2.08	0.11	–1.51, 1.74
Gender								
Girl	Ref.	Ref.	Ref.	Ref.	Ref.	Ref.	Ref.	Ref.
Boy	–2.88*	–5.25, –0.51	–1.58	–4.00, 0.84	4.79***	–7.15, –2.42	–3.29**	–5.63, –0.95
Race/Ethnicity								
African American	Ref.	Ref.	Ref.	Ref.	Ref.	Ref.	Ref.	Ref.
Hispanic	1.89	–0.84, 4.61	1.69	–0.89, 4.27	0.07	–2.83, 2.97	–0.37	–2.87, 2.14
Another race	3.21	–0.85, 7.27	1.12	–2.78, 5.02	3.69	–0.39, 7.77	0.98	–2.61, 4.58
Sexual orientation								
Gay/lesbian/bisexual/other	Ref.	Ref.	Ref.	Ref.	Ref.	Ref.	Ref.	Ref.
Heterosexual	–3.97**	–6.82, –1.13	–2.40	–5.31, 0.51	5.97***	–8.69, 13.67	–3.87	–6.58, –1.15

*Note*. Coef. = unadjusted coefficient; β = standardized beta; 95% CI = 95% confidence interval; Ref. = reference group. Sample sizes vary due to missing data.

Statistical significance at *p < .05. **p < .01. ***p < .001.

## Discussion

This study developed a 10-item age-appropriate HD measure and assessed its concurrent validity concerning DV perpetration, lifetime substance use, and mental health among sixth graders. Each item in the HD measure elicited responses underscoring their relevance among our study population age group. Our results also demonstrated significant associations between HD and most of the outcome variables, except for lifetime drug use, providing evidence of concurrent validity.

Existing ACE measures have been primarily developed for adults (Afifi, Taillieu, et al., 2020) and may not be appropriate for utilization among younger adolescents, including sixth graders. The existing ACE measures for adults have demonstrated evidence of construct validity, internal reliability, and structural validity (Afifi, Salmon, et al., 2020; Ford et al., 2014; Mei et al., 2022; Mersky et al., 2017). Conversely, there is a lack of domain-specific measures, which lack any form of psychometric testing, especially among middle schoolers.

Results from this study suggest that the newly developed HD measure is appropriate for youth. Notably, all items in our HD measure were endorsed by our study population, including those not commonly tested. Existing literature suggests that parental separation/divorce is the most prevalent HD exposure (Afifi, Taillieu, et al., 2020; Crouch et al., 2019; Turney, 2018; Turney & Olsen, 2019). Our findings were consistent with the literature, as the most common type of HD exposure in our study sample was parental separation/divorce (33.3%). In addition, some of the HD exposures in our sample were relatively higher compared to existing literature, including parental death (17.5%). These findings suggest that the measure includes HD items relevant to sixth graders and produced results that were somewhat consistent with previous studies that relied on more adult-oriented language.

Findings from our study also provide strong evidence of concurrent validity. Consistent with existing literature, we found significant associations between HD and both DV perpetration outcomes (Foshee et al., 2015; Temple et al., 2013). The existing studies reported weaker associations between HD (particularly witnessing interparental violence and family conflict) and physical and psychological DV perpetration compared to our study findings. The stronger associations in our study may be because our measure included several age-appropriate HD items. This allowed us to account for multiple aspects of HD, rather than just a single aspect, which may have contributed to larger effects.

Our results also exhibited a more robust effect between HD and alcohol use, compared to other studies (Chatterjee et al., 2018; McCoy et al., 2020). However, our nonsignificant finding regarding HD and drug use may have been due to the limited sample size or because this exposure was the least prevalent. The mental health findings were also consistent with previous literature indicating a direct association between HD and depression and anxiety (Wang et al., 2021).

Notably, previous studies examining associations between HD and key outcomes limited their HD assessment to one to three items (Chatterjee et al., 2018; Foshee et al., 2015; Temple et al., 2013; Wang et al., 2021). Therefore, some aspects of HD were not examined in previous studies, which could further limit our understanding of HD and the potential cumulative effects of different exposures. The 10-item HD measure was designed to capture the different aspects of the HD construct. Moreover, our study population endorsed all items in the HD measure, which further demonstrates the importance of including these additional elements in HD measures to better understand the magnitude of the effect of HD on these key outcomes.

Our study population consists predominantly of Hispanic adolescents, with a significant portion identifying as sexual minorities. This underscores the importance of considering the unique social and cultural contexts that shape the experiences of HD. While these findings may be relevant to similar populations, they may not be fully generalizable to groups with different racial/ethnic or sexual orientation distributions. Furthermore, the regional context of Southeast Texas likely reflects specific cultural and socioeconomic factors that influence HD experiences. Future research is essential to replicate and expand these findings in larger and more diverse populations across various geographic settings. Such efforts are critical for developing a more comprehensive understanding of HD and its impacts across different populations.

### Strengths and Limitations

To our knowledge, this is the first study to develop an age-appropriate HD measure for middle schoolers. We used a well-accepted measure development approach, and our HD measure is informed by existing ACE measures and their HD items, which have demonstrated evidence of validity (Afifi, Salmon, et al., 2020; BRFSS, 2021; DeVellis, 2011; Ford et al., 2014; Holden et al., 2020; Koita et al., 2018; Mersky et al., 2017; NSCH, 2021; PEARLS, 2019; Thakur et al., 2020; Weller et al., 2022). Our HD measure is also pragmatic as it includes a reasonable number of items to capture important aspects of HD while being concise enough to be included in surveys distributed to adolescents in school settings. Furthermore, it may be beneficial to use the HD measure as an emergent screening tool among younger adolescents in clinic settings to help increase recognition in addressing their behavioral and mental health issues impacted by HD. Finally, we tested multiple outcomes when examining the concurrent validity to ensure our HD measure exhibited expected associations in this age group, and our findings supported concurrent validity across all the tested outcomes except for lifetime drug use.

There are several limitations to this study. First, we focused our assessment on concurrent validity, which is an important form of criterion-related validity (Bandalos, 2018). However, additional reliability and validity testing is required to further understand other psychometric properties of the measure. Second, we acknowledge the importance of further pilot testing with larger samples to assess the HD measure’s sensitivity and refine its performance. Third, we had a limited sample size, which may have impacted our power to detect a significant effect regarding the association between HD and lifetime drug use. Finally, we categorized adolescents who never dated or did not have any dating partners in the past year as non-perpetrators, though other studies have used a similar categorization (Peskin et al., 2019; Wolfe et al., 2009).

## Conclusion

There is an important need for age-appropriate HD measures, especially for adolescents. We developed a measure for middle schoolers that includes ten HD items, better capturing the different aspects of HD. Our findings suggest the measure is appropriate for early adolescents, pragmatic, and has strong evidence of concurrent validity by revealing expected associations between HD and the multiple outcomes assessed in our study. This measure may be helpful for assessing HD among middle schoolers both in school and clinic settings so that preventive efforts can be implemented to mitigate HD-related consequences.

## Supplemental Material

sj-docx-1-jiv-10.1177_08862605251341285 – Supplemental material for Development of an Age-Appropriate Household Dysfunction Measure and its Concurrent Validity With Multiple Outcomes Among Middle School Adolescents in Southeast Texas Supplemental material, sj-docx-1-jiv-10.1177_08862605251341285 for Development of an Age-Appropriate Household Dysfunction Measure and its Concurrent Validity With Multiple Outcomes Among Middle School Adolescents in Southeast Texas by Sumaita Choudhury, Timothy J. Walker, Emily T. Hébert, Christine M. Markham, Ross Shegog, Robert C. Addy and Melissa F. Peskin in Journal of Interpersonal Violence

sj-docx-2-jiv-10.1177_08862605251341285 – Supplemental material for Development of an Age-Appropriate Household Dysfunction Measure and its Concurrent Validity With Multiple Outcomes Among Middle School Adolescents in Southeast Texas Supplemental material, sj-docx-2-jiv-10.1177_08862605251341285 for Development of an Age-Appropriate Household Dysfunction Measure and its Concurrent Validity With Multiple Outcomes Among Middle School Adolescents in Southeast Texas by Sumaita Choudhury, Timothy J. Walker, Emily T. Hébert, Christine M. Markham, Ross Shegog, Robert C. Addy and Melissa F. Peskin in Journal of Interpersonal Violence

sj-docx-3-jiv-10.1177_08862605251341285 – Supplemental material for Development of an Age-Appropriate Household Dysfunction Measure and its Concurrent Validity With Multiple Outcomes Among Middle School Adolescents in Southeast Texas Supplemental material, sj-docx-3-jiv-10.1177_08862605251341285 for Development of an Age-Appropriate Household Dysfunction Measure and its Concurrent Validity With Multiple Outcomes Among Middle School Adolescents in Southeast Texas by Sumaita Choudhury, Timothy J. Walker, Emily T. Hébert, Christine M. Markham, Ross Shegog, Robert C. Addy and Melissa F. Peskin in Journal of Interpersonal Violence

sj-docx-4-jiv-10.1177_08862605251341285 – Supplemental material for Development of an Age-Appropriate Household Dysfunction Measure and its Concurrent Validity With Multiple Outcomes Among Middle School Adolescents in Southeast Texas Supplemental material, sj-docx-4-jiv-10.1177_08862605251341285 for Development of an Age-Appropriate Household Dysfunction Measure and its Concurrent Validity With Multiple Outcomes Among Middle School Adolescents in Southeast Texas by Sumaita Choudhury, Timothy J. Walker, Emily T. Hébert, Christine M. Markham, Ross Shegog, Robert C. Addy and Melissa F. Peskin in Journal of Interpersonal Violence
