# Supplementary material for: Development of an Age-Appropriate Household Dysfunction Measure and its Concurrent Validity With Multiple Outcomes Among Middle School Adolescents in Southeast Texas
Source: J Interpers Violence. 2025 May 29;41(11-12):3751–71. doi: 10.1177/08862605251341285 (PMC13139674; doi:10.1177/08862605251341285)
Supplement: sj-docx-1-jiv-10.1177_08862605251341285 – Supplemental material for Development of an Age-Appropriate Household Dysfunction Measure and its Concurrent Validity With Multiple Outcomes Among Middle School Adolescents in Southeast Texas [file sj-docx-1-jiv-10.1177_08862605251341285.docx]

# **Supplement**

##### **Supplement 1: HD Measure**

| **HD Measure** |
| --- |
| 1. Do you live with anyone who is sad the majority of the time? |
| 1. Do you live with anyone who drinks too much? |
| 1. Do you live with anyone who abuses drugs or their medications? |
| 1. Do you live with anyone who went to jail or is currently in jail? |
| 1. Are your parents separated or divorced? |
| 1. Do you live with anyone who says mean things or hurts other members in your house? |
| 1. Have you lived with a parent or guardian who died? |
| 1. Is your family having problems with stable housing? (For example, not having a permanent place to live, having to move often, or having to live with multiple family members). |
| 1. Do you often worry that you do not have enough food to eat at home? |
| 1. Do you live with anyone who has a serious physical illness or disability? |
