# Supplementary material for: Development of an Age-Appropriate Household Dysfunction Measure and its Concurrent Validity With Multiple Outcomes Among Middle School Adolescents in Southeast Texas
Source: J Interpers Violence. 2025 May 29;41(11-12):3751–71. doi: 10.1177/08862605251341285 (PMC13139674; doi:10.1177/08862605251341285)
Supplement: sj-docx-2-jiv-10.1177_08862605251341285 – Supplemental material for Development of an Age-Appropriate Household Dysfunction Measure and its Concurrent Validity With Multiple Outcomes Among Middle School Adolescents in Southeast Texas [file sj-docx-2-jiv-10.1177_08862605251341285.docx]

# **Supplement**

##### **Supplement 2: Physical and Psychological DV Perpetration Measures**

| **CADRI DV Subscales** | **Items** |
| --- | --- |
| **Instruction:** The following questions ask about things that you may have ever done toward a boyfriend or girlfriend (someone that you have dated, gone out with, gone steady with). Please mark YES or NO for each question. Remember, all of your answers will be kept private, and no one will know your answers. | |
| Physical | 1. I threw something at him/her. 2. I kicked, hit, or punched him/her. 3. I slapped him/her or pulled his/her hair. 4. I pushed, shoved, or shook him/her. |
| Psychological | 1. I did something to make him/her jealous. 2. I brought up something bad that he/she had done in the past. 3. I said things just to make him/her angry. 4. I spoke to him/her in a hostile or mean tone of voice. 5. I insulted him/her with put-downs. 6. I made fun of him/her in front of others. 7. I kept track of who he/she was with and where he/she was. 8. I blamed him/her for the problem. 9. I accused him/her of flirting with another girl/guy. 10. I threatened to end the relationship. 11. I tried to turn my friends against him/her. 12. I said things to his friends about him/her to turn them against him/her. 13. I spread rumors about him/her. |
