# Supplementary material for: Development of an Age-Appropriate Household Dysfunction Measure and its Concurrent Validity With Multiple Outcomes Among Middle School Adolescents in Southeast Texas
Source: J Interpers Violence. 2025 May 29;41(11-12):3751–71. doi: 10.1177/08862605251341285 (PMC13139674; doi:10.1177/08862605251341285)
Supplement: sj-docx-3-jiv-10.1177_08862605251341285 – Supplemental material for Development of an Age-Appropriate Household Dysfunction Measure and its Concurrent Validity With Multiple Outcomes Among Middle School Adolescents in Southeast Texas [file sj-docx-3-jiv-10.1177_08862605251341285.docx]

# **Supplement**

##### **Supplement 3: Depression Measure**

| **Depression Measure** |
| --- |
| **Instruction:** Now, these next questions ask about thoughts and feelings.  Please think about each statement. Remember, there are no right or wrong answers, and all your answers will be kept private. In the past 30 days, how often… |
| 1. Were you very sad? |
| 1. Were you grouchy or irritable, or in a bad mood? |
| 1. Did you feel hopeless about the future? |
| 1. Did you feel like not eating, or feel like eating more than usual? |
| 1. Did you sleep a lot more or a lot less than usual? |
| 1. Did you have difficulty concentrating on your schoolwork? |
