# Supplementary material for: Development of an Age-Appropriate Household Dysfunction Measure and its Concurrent Validity With Multiple Outcomes Among Middle School Adolescents in Southeast Texas
Source: J Interpers Violence. 2025 May 29;41(11-12):3751–71. doi: 10.1177/08862605251341285 (PMC13139674; doi:10.1177/08862605251341285)
Supplement: sj-docx-4-jiv-10.1177_08862605251341285 – Supplemental material for Development of an Age-Appropriate Household Dysfunction Measure and its Concurrent Validity With Multiple Outcomes Among Middle School Adolescents in Southeast Texas [file sj-docx-4-jiv-10.1177_08862605251341285.docx]

# **Supplement**

##### **Supplement 4: Anxiety Measure (GAD-7)**

| **Anxiety Measure** |
| --- |
| **Instruction:** Now, think about the last two weeks. Over the last 2 weeks, how often have you been bothered by the following problems? |
| 1. Feeling nervous, anxious or on edge |
| 1. Not being able to stop or control worrying |
| 1. Worrying too much about different things |
| 1. Trouble relaxing |
| 1. Being so restless that it is hard to sit still |
| 1. Becoming easily annoyed or irritable |
| 1. Feeling as if something awful might happen |
